# Environmental and health impacts of using food waste as animal feed: a comparative analysis of food waste management options

**DOI:** 10.1016/j.jclepro.2016.05.049

**Published:** 2017-01-01

**Authors:** Ramy Salemdeeb, Erasmus K.H.J. zu Ermgassen, Mi Hyung Kim, Andrew Balmford, Abir Al-Tabbaa

**Affiliations:** a Department of Engineering, University of Cambridge, Trumpington Street, Cambridge CB2 1PZ, UK; b Conservation Science Group, Department of Zoology, University of Cambridge, The David Attenborough Building, Pembroke Street, Cambridge CB2 3QZ, UK; c Department of Environmental Planning, Graduate School of Environmental Studies, Seoul National University, San 56-1, Sillim-Dong, Gwanak-Gu, Seoul 151-742, South Korea

**Keywords:** Food waste, Hybrid life-cycle assessment, Animal feed, Anaerobic digestion, Composting, Swill

## Abstract

The disposal of food waste is a large environmental problem. In the United Kingdom (UK), approximately 15 million tonnes of food are wasted each year, mostly disposed of in landfill, via composting, or anaerobic digestion (AD). European Union (EU) guidelines state that food waste should preferentially be used as animal feed though for most food waste this practice is currently illegal, because of disease control concerns. Interest in the potential diversion of food waste for animal feed is however growing, with a number of East Asian states offering working examples of safe food waste recycling – based on tight regulation and rendering food waste safe through heat treatment. This study investigates the potential benefits of diverting food waste for pig feed in the UK. A hybrid, consequential life cycle assessment (LCA) was conducted to compare the environmental and health impacts of four technologies for food waste processing: two technologies of South Korean style-animal feed production (as a wet pig feed and a dry pig feed) were compared with two widespread UK disposal technologies: AD and composting. Results of 14 mid-point impact categories show that the processing of food waste as a wet pig feed and a dry pig feed have the best and second-best scores, respectively, for 13/14 and 12/14 environmental and health impacts. The low impact of food waste feed stems in large part from its substitution of conventional feed, the production of which has substantial environmental and health impacts. While the re-legalisation of the use of food waste as pig feed could offer environmental and public health benefits, this will require support from policy makers, the public, and the pig industry, as well as investment in separated food waste collection which currently occurs in only a minority of regions.

## Highlights

• There is growing interest in the re-legalisation of food waste as animal feed in the EU.
• We compare environmental impacts of 4 food waste disposal options, including as pig feed.
• We use a hybrid LCA approach to overcome the limitations of conventional LCA.
• Recycling food waste as pig feed has lower environmental impact than AD or composting.

## 1 Introduction

The disposal of food waste poses a large environmental problem. Food waste is abundant: in the UK, approximately 15 million tonnes are wasted annually (234 kg/person/year or 50% of food) (WRAP, 2015) and the available disposal options each have substantial environmental impacts. Landfilling produces large quantities of greenhouse gases (GHG) and is therefore being phased out under new EU regulation (EC, 2014), but is still the destination of up to 48% of food waste in parts of the UK (House of Lords, 2014). Incineration and composting also produce greenhouse gases, and wastewater from anaerobic digestion causes eutrophication and acidification of local ecosystems (Evangelisti et al., 2014, Salemdeeb and Al-Tabbaa, 2015, Whiting and Azapagic, 2014).

**Fig. 1:**
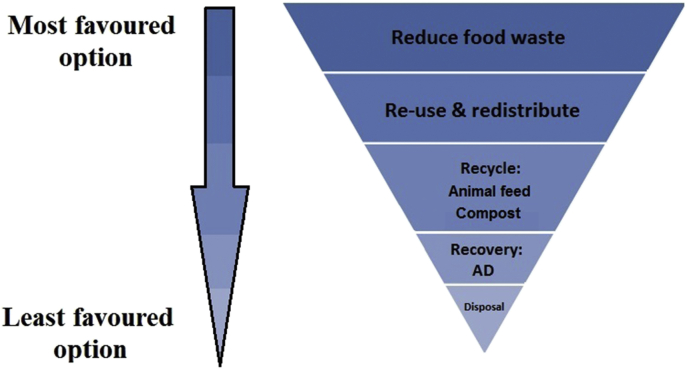
The food waste hierarchy. Adapted from Papargyropoulou et al. (2014) and zu Ermgassen et al. (2016). AD = anaerobic digestion.

To aid the selection of food waste disposal technologies, the EU provides guidelines on which disposal technologies are preferable (EC, 2014). This so-called food waste hierarchy (Fig. 1), stipulates that governments should prioritise efforts (in order of most to least preferable) to (i) reduce food waste, (ii) redistribute it (e.g. to the homeless), (iii) recycle it as animal feed and (iv) compost, (v) recover energy through anaerobic digestion, and finally, (vi) landfill the remainder. This legislation is, however, notably not applied with respect to the use of food waste as animal feed, because it is currently illegal to use most food waste as feed in the EU.

Though food waste is the archetypal pig feed, if it contains meat wastes and is not heat-treated it can transmit diseases, such as foot-and-mouth disease and African swine fever. In 2001, a UK farmer illegally fed uncooked food waste to pigs, precipitating the foot-and-mouth disease epidemic, which cost the UK economy £8 billion (UK House of Commons report, 2002). As a result, the recycling of food waste as animal feed was banned across the EU (EC, 2002). The law still permits the feeding of some food wastes where it can be demonstrated that there is no risk of contamination with animal products, but this represents only a small proportion of all EU food waste. Currently, of the 89–100 million tonnes of food waste produced in the EU per year (Monier et al., 2010), only around 3 million tonnes are recycled as animal feed (zu Ermgassen et al., 2016).

In other parts of the world, however, food waste continues to be commonly used as animal feed, including in modern systems of pig production. Heat treatment renders food waste safe for animal feed (Edwards, 2000, Garcıa et al., 2005, OIE, 2009), and in nations such as Japan and South Korea 35.9% and 42.5%, respectively, of food waste is recycled as feed. There, the use of food waste is closely regulated: legislation governs the heat treatment, storage, and transport of food waste feed (Sugiura et al., 2009, zu Ermgassen et al., 2016).

Amid increases and volatility in the price of conventional feed (AHDB Market Intelligence, 2013, AHDB Market Intelligence, 2006), and concerns about the environmental impact of grain- and soybean-based feeds (Nguyen et al., 2012), there is growing interest in the potential relegalisation and promotion of the use of food waste as pig feed (The Economist, 2013, The Pig Idea, 2014). A recent survey of 1195 animal feed practitioners (from industry, academia, and NGOs) identified the use of food waste as a priority research area for sustainable animal nutrition (Makkar and Ankers, 2014).

In this study we evaluate the environmental and health impacts of converting municipal food wastes into pig feed in the UK. We conducted a hybrid life cycle assessment (LCA) to compare the environmental impacts of two technologies for recycling municipal food waste as animal feed (as a dry or a wet pig feed), with two well established food waste management options: composting and anaerobic digestion (DEFRA, 2015a). In doing so, we address a gap in the literature. Few previous studies have evaluated the potential for recycling food waste as animal feed in the EU, even fewer consider environmental impacts other than greenhouse gas emissions or land use and, none, to the author's knowledge, have thus far specifically considered the use of municipal food wastes as animal feed. zu Ermgassen et al. (2016) suggest that if the EU were to recycle food waste as pig feed at similar rates to nations such as Japan and South Korea, this would provide enough feed to support 20% of EU pork production, reducing the land use of EU pork by 1.8 million hectares of farmland. Four European studies have evaluated environmental impacts beyond land use, though these considered only manufacturing or retail food wastes or agricultural co-products (such as beet tails or soybean meal) (Tufvesson et al., 2013, Vandermeersch et al., 2014, van Zanten et al., 2014, Eriksson et al., 2015). These studies each adopted a bottom-up life cycle assessment approach and therefore have several inherent drawbacks that lead to system incompleteness and underestimate environmental impacts (Bernstad and la Cour Jansen, 2012, Laurent et al., 2014a, Laurent et al., 2014b). We overcome these methodological limitations by taking a more holistic, hybrid LCA approach (described in more detail below). This study focuses on municipal food wastes because they make up 66% of EU food waste (Monier et al., 2010) and are suitable for animal feed – they are currently used in both South Korea and China (Chen et al., 2015, Stuart, 2009) and have historically been used in the EU (Fairlie, 2010).

## 2 Material and methods

We evaluated the environmental and health impacts of processing 1 tonne of municipal food waste in the UK using four different technologies: (a) conversion into dry pig feed, (b) conversion into wet pig feed, (c) anaerobic digestion, and (d) composting (Table 1). We used a hybrid, consequential life cycle approach, expanding the system boundary of the analysis to take into consideration the substituted processes. Product substitution operates as follows: if food waste is processed to produce dry pig feed, for example, this will lead to avoided emissions from the substitution of conventional pig feed, but also knock-on emissions from the composting or anaerobic digestion that did not take place. Similarly, the total emissions from composting are the sum of the emissions released during composting, minus the emissions from the production of fertiliser which compost replaces, plus the additional emissions from the conventional pig feed and electricity production, which result from the food waste not being recycled as pig feed or anaerobically digested.

The hybrid LCA approach combines conventional process-based LCA and an input–output based LCA (Salemdeeb and Al-Tabbaa, submitted for publication). This approach is used to counter the limitations of conventional LCAs, which face a truncation problem: system boundaries are set *a priori* and typically cut off part of the product life cycle for the sake of simplicity (Bullard et al., 1978, Lenzen, 2001). Input–output approaches use data on the total project cost to estimate upstream-processes that are not modelled using traditional LCA, such as the manufacture of electronic products or technical consulting services, and thereby mitigate truncation error. The input–output component of the hybrid model was a single region model with a domestic technology assumption (i.e. economic activities in the country of origin of imports are the same as in the importing country; Appendix 1). The LCA component of the analysis was conducted in EASETECH, a LCA tool developed at the Technical University of Denmark (Clavreul et al., 2014).

We characterised and normalised results for 14 mid-point impact categories (detailed in Table 1) for each of our four food waste recycling technologies; these impact categories include a diverse set of environmental and human health indicators to give a multi-criteria assessment of the impacts of our four food waste disposal technologies. Characterisation involves the calculation of each impact (for example, global warming potential requires the weighting of impacts from emissions of carbon dioxide, nitrous oxides and methane). Normalisation then permits comparison of the relative importance of each impact category, by expressing the process' emissions as a proportion of the total emissions (per capita) in the EU-27 in 2010. The global warming potential and particulate matter emissions from recycling 1 tonne of food waste are, for example, scaled relative to the per capita greenhouse gas and particulate matter emissions in the year 2010 (and are reported in units of milli-Person equivalents, mPE). Characterisation and normalisation followed ILCD methods (Benini et al., 2014, JRC, 2010).

### 2.1 Food waste disposal technologies

**Fig. 2:**
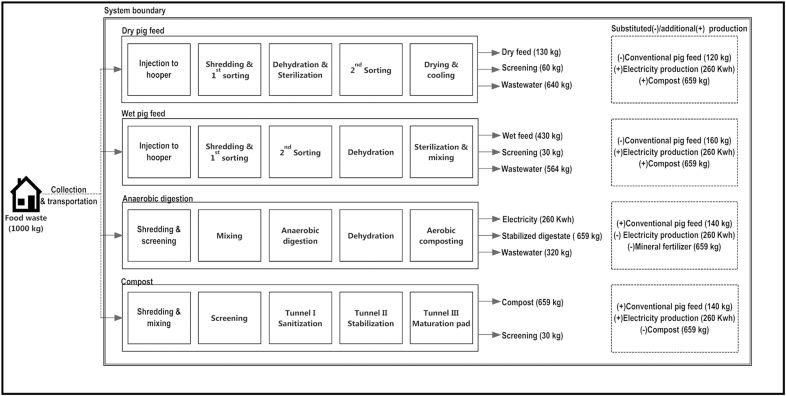
Steps involved in the processing of food waste by the four food waste disposal technologies. Only major material flows are shown: minor inputs (e.g. water, corn in the case of wet feed) and evaporation are not included for the sake of clarity. Outputs are indicated by arrows and substituted products are shown in the boxes on the right-hand side. Mineral fertilizer substitution rates of digestate and compost are listed in section 2.1.3 and Appendix 3, respectively.

The four food waste disposal technologies and substituted products are depicted in Fig. 2. As all technologies require separate collection of food waste, food waste collection and transportation are excluded from this study. Food waste packaging is also excluded due to its insignificant impact (Lebersorger and Schneider, 2011).

#### 2.1.1 Dry pig feed

As the use of municipal food waste as animal feed is illegal in the EU, we used process-specific data from factories producing food waste feed in South Korea (Kim and Kim, 2010), where there were 259 registered feed manufacturers as of 2010 (Ministry of Environment, 2010).

Food waste is loaded into a hopper, shredded and filtered for contaminants (Fig. 2). It is then sterilised and dehydrated by air-drying at 390 °C. Under South Korean law, food waste must be heat treated to a core temperature of >80 °C for a minimum of 30 min (National Institute of Environmental Research, 2012); in comparison, before the ban on using food waste as animal feed, EU law used to mandate heating food waste to 100 °C for 60 min (Stuart, 2009). The feed is sorted again before one more step of drying, producing 140 kg of dry feed per tonne of food waste (with a moisture content of 21.8%, i.e. 109.5 kg of feed on a dry matter basis).

The food waste feed substitutes conventional feed 1:1 on a dry matter basis (zu Ermgassen et al., 2016). The ingredients of the substituted conventional feed (Appendix 2) are based on the weighted mean feed intake of all pigs in the pork production life cycle (sows, piglets, and slaughter pigs), taken from an LCA of UK pork production (Stephen, 2012). The impact of feed ingredients that are co-products was allocated according to their economic value. Soybeans, for example, are processed into both soybean meal, a common pig feed ingredient, and soy oil; soybean meal makes up 60% of soybean value, and soy oil the other 40% (USDA, 2012), and so 60% of the impact of soybean production was allocated to soybean meal. When calculating the environmental impact of soybean meal we use the most recent available inventory data on soybean production in Brazil (Nemecek et al., 2014); Brazil is the source of 88% of the UK soybean supply (FAO, 2014).

In using data from South Korean processing plants, we assume that the municipal food waste used to generate animal feed in South Korea is comparable with municipal food waste in the UK. To check this assumption, we compared data for South Korean food waste with UK data, and found that the compositions are broadly similar (Table 2).

#### 2.1.2 Wet pig feed

When food waste is used as wet pig feed, it is injected into the hopper, shredded, and twice filtered for contaminants (Fig. 2). It is then partially dehydrated and heat-treated to 100 °C to sterilise it. It is mixed with 25 kg of ground maize before storage, to produce 430 kg of wet feed per tonne of food waste, with a mean dry matter content of 30.9%. The substitution of conventional feed is calculated as for dry feed.

#### 2.1.3 Anaerobic digestion

In this process, food waste is shredded, sieved, and sent to a digestion tank. The digestate has a dry solids content between 25 and 40% and is digested at a temperature between 50 and 55 °C (Hall et al., 2014). AD digestate utilization efficiencies are presented in Table 3. Biogas is then collected, purified and used to generate electricity (260 kwh/tonne of processed food waste), which substitutes electricity produced from the UK energy mix (Table 4). Finally, the remaining digestate undergoes dewatering and refinement producing a high-quality AD cake, which substitutes nitrogen, phosphorous and potassium fertilisers with an efficiency of 34.5%, 46% and 60%, respectively. Benefits from the contribution made by sulphur, magnesium, and other organic compounds in compost are excluded (Wallace, 2011).

#### 2.1.4 Composting

Incoming waste is shredded, mixed and aerated for 14–21 days at a minimum temperature of 60 °C for 48 h (Hall et al., 2014). The compost is then stored in windrows for a 56 day maturation phase. The matured material is removed, screened, and packed as compost. The compost utilization efficiencies used are: 20% for N, 100% for P, and 100% for K (Andersen et al., 2010) and the compost is considered to be applied on loam soil (see Appendix 3), where it substitutes synthetic fertilizers on a 1:1 basis. The composting process is assumed to be well managed, i.e. no failures occur that give rise to high emissions of methane and other products of anaerobic conditions. All leachate water is reused during the composting process for re-wetting in the reception area and the maturation pad.

### 2.2 Sensitivity analysis

A three-step sensitivity analysis approach based on Clavreul et al. (2012) was conducted to evaluate the level of uncertainty in our results. First, the stages with the highest environmental burdens were identified using a hotspot analysis. Then a perturbation analysis was conducted on stages identified in the previous step: we calculated the sensitivity ratio (Eq. (1)) for all parameters, by varying each parameter by ±10%.

(1) $$Sensitivity\;Ratio\left(SR\right)=\frac{\frac{\Delta result}{Initial\;result}}{\frac{\Delta parameter}{Initial\;parameter}}$$

For each of our four food waste disposal technologies, we selected the eight parameters which had the highest sensitivity ratio, assigned them probability distributions and performed a Monte Carlo analysis (1000 simulations) to generate confidence intervals for our results. The selected parameters and probability distributions are listed in Appendix 4. For each metric, we tested for the significance of differences between technologies, also using Monte Carlo methods. We randomly sampled estimates of the mean for each technology, and calculated the difference between each technology, repeating this resampling 1000 times. We then tested to see if the difference between technologies overlapped with zero at the 99% confidence level.

Each technology was then ranked (1–4; 1 = best, 4 = worst) for each of our 14 mid-point metrics and the mean ranking for each technology was calculated.

### 2.3 Life cycle inventory data

The hybrid LCA analysis requires two datasets: process-based physical data, and input–output monetary data.

#### 2.3.1 Physical data

Data, listed in Table 5, was either compiled or calculated based on information from project documents, literature, and the WRATE database (Hall et al., 2014). Upstream and downstream material flows and emissions were collected using existing databases, primarily the Swiss Ecoinvent database v2.2 (Ecoinvent, 2014). These processes include the acquisition of raw material and energy, production, on-site operation, and waste disposal (i.e. cradle to grave).

#### 2.3.2 Monetary data

Monetary data were obtained from two sources: data of animal feed technologies were obtained by direct communication with the Korean Ministry of Environment; expenditure data for 2014, available in South Korean Won (₩), was converted into British pound using purchasing power parity coefficients of the year 2014 (Appendix 5). Data of both AD and composting were obtained from UK industrial partners (Appendix 6). Appendix 1 lists sources of data and components for the IO-based element of the hybrid approach.

## 3 Results and discussion

**Fig. 3:**
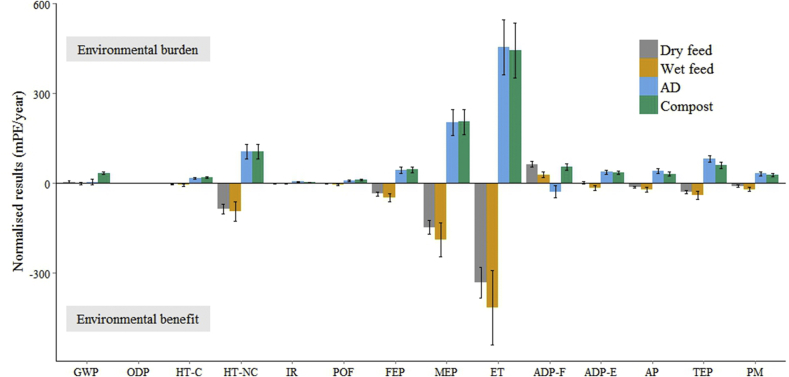
Normalised environmental and health impacts of four recycling technologies for food waste: dry animal feed, wet animal feed, anaerobic digestion (AD), and composting. Units (mPE) relate a process' emissions to per capita emissions in the EU in 2010. GWP = global warming potential; ODP = ozone depletion; HT-C = emissions of carcinogens; HT-NC = emissions of non-carcinogenic toxins; IR = ionising radiation; POF = photochemical oxidant formation; FEP = freshwater eutrophication; MEP = marine eutrophication; ET = ecotoxicity; ADP-F = depletion of fossil fuels; ADP-E = depletion of non-fossil fuel abiotic resources; AP = acidification; TEP = terrestrial eutrophication, PM = particulate matter emissions. Error bars show one standard deviation.

The recycling of food waste as wet pig feed had the best score for 13 of 14 environmental and health impacts while dry feed had the second-best score for 12 of 14 impacts (Fig. 3 and Table 6). The mean ranking of the four technologies (1 = best, 4 = worst) were wet feed: 1.1, dry feed: 2.2, AD: 3.3, and composting: 3.4. Composting had the worst score for seven environmental indicators, anaerobic digestion the worst score for eight (including two joint-worst scores shared with composting), and dry feeding the worst score for one indicator (depletion of fossil fuels).

After normalisation, composting and anaerobic digestion had disproportionate impacts through eutrophication (terrestrial, marine, and freshwater), environmental toxicity (including non-carcinogenic toxicity, NC-HT, and ecotoxicity, ET), and acidification (Fig. 3). The superiority of wet and dry feed in these impact categories stems in large part from their substitution of conventional animal feed (Appendix 7). All stages of conventional feed production, including the farming and transport of raw materials to the feed processing centre, the milling of the feed, and the storage of the feed mixes, contribute to the substantial emissions in these impact categories. For example, hotspot analysis shows that freshwater eutrophication impacts are principally caused by the use of phosphate-based fertilisers in the farming of feed crops, and marine eutrophication is principally caused by the energy consumption and fuel inputs involved in shipping feed ingredients (such as soybean meal).

For non-carcinogenic toxicity, we found that the concentration of zinc during the growth of rapeseed (an ingredient in conventional pig feed) accounts for nearly 35% of the impact. This result agrees with other studies highlighting concerns that rapeseed may contain high concentrations of heavy metals (such as zinc and copper) and allergens. Heavy metals from the soil are known to accumulate in the roots, plant, and seeds of rapeseed (van der Spiegel et al., 2013).

Our results support the diversion of food waste to animal feed, before composting or anaerobic digestion, as proposed under the food waste hierarchy. The difference between AD and composting is however less clear: composting rated better than AD for 7/14 indicators, including acidification, terrestrial eutrophication, and particulate matter; AD rated better for 5/14 indicators, including greenhouse gas emissions and ozone depletion, and there was no significant difference between them for 2/14 indicators (marine eutrophication and non-carcinogenic toxicity).

### 3.1 Comparison with previous literature

**Fig. 4:**
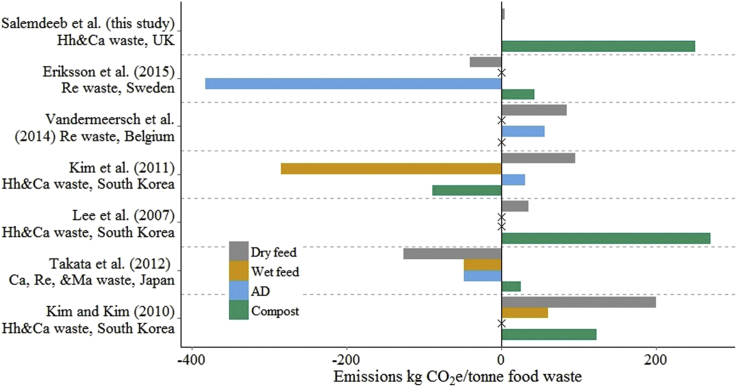
Results of seven LCA studies reporting the greenhouse gas emissions per tonne of food waste. The location of and waste stream evaluated in each study (Hh = household, Ca = catering, Re = retail, and Ma = manufacturing food waste) are listed. Crosses are marked where a study did not include a technology in their analysis. Where a study reported emissions for multiple food waste types (e.g. meat or banana wastes from supermarkets), the mean emissions are shown. Two further LCA studies (Ogino et al., 2012, Tufvesson et al., 2013) use different functional units (reporting results per kg of animal feed and per MJ of fuel energy, rather than per tonne of food waste) and so cannot be displayed for comparison. AD = anaerobic digestion.

While these results suggest that the re-legalisation of the use of municipal food waste as animal feed has potential to reduce the impact of food waste disposal in the UK, LCA results are often location- and assumption-dependent (Bernstad and la Cour Jansen, 2012). We therefore compare our results with previous LCAs of food waste recycling. Previous studies did not evaluate the same portfolio of environmental indicators as this study, but greenhouse gas consequences have been calculated for nine studies, shown in Fig. 4. Though the exact figures vary substantially between studies, some broad patterns emerge. Wet feed has lower emissions than AD (2/3 studies making this comparison); AD produces lower emissions than dry feed (4/5 studies); and wet and dry feed produce lower emissions than composting (4/4 and 4/6 studies, respectively). Some of the differences between studies may be due to particularities of the locations where the studies were performed and the waste stream analysed. Only two of these studies evaluated food waste recycling in Europe (Eriksson et al., 2015, Vandermeersch et al., 2014), and neither (as here) looked at municipal food waste (instead evaluating retail waste).

Study assumptions also explain some of the differences: none of the studies in Fig. 4 include land use change and they therefore underestimate the avoided emissions from animal feed substitution. This truncated-boundary problem underestimates the GHG emissions from animal feed ingredients by up to nine-times (van Middelaar et al., 2013). For example, Eriksson et al. use a GHG emission for soybean meal of 0.66 kgCO_2_e/kg, while our study uses the most recent figure of 4.4 kgCO_2_e/kg (Nemecek et al., 2014). Eriksson et al. also report large avoided greenhouse gas emissions when food waste is anaerobically digested compared with its use as dry animal feed (−381.4 vs −40.84 kgCO_2_e/kg; figures for this study: 3.80 vs 3.96 kgCO_2_e/kg). This difference stems from assumptions about the yields of biogas during anaerobic digestion and the energy mix substituted. Eriksson et al. assume that the entire theoretical yield of biogas was produced, while our work is based on an actual AD plant figures; their study assumes biogas replaces diesel as a fuel for city buses, while this study assumes biogas substitutes UK electricity production (natural gas 61.46% and coal 38.54%).

### 3.2 Robustness of results

To better understand the uncertainty in our results, we tested the sensitivity of our results to the parameter values chosen in the model. Despite the large variability in some parameters (Appendix 4), the indicator values for all metrics are significantly different from one another (p < 0.01), except for the effect of composting and anaerobic digestion on marine eutrophication and non-carcinogenic toxicity.

**Fig. 5:**
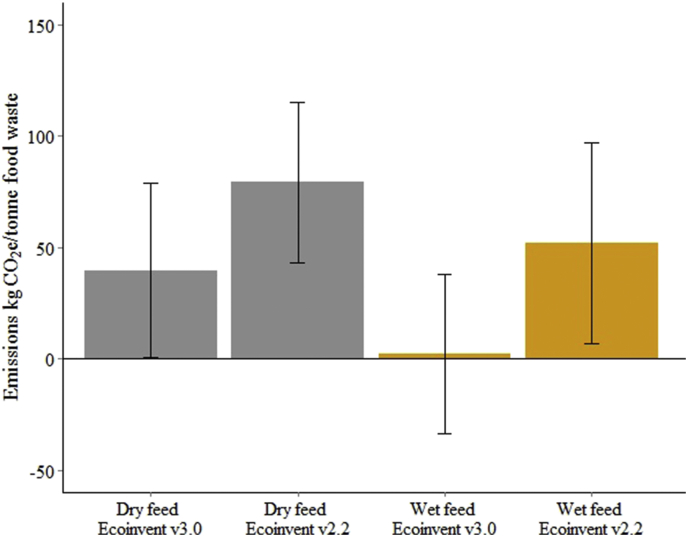
Greenhouse gas emissions from using food waste as dry feed and wet feed, comparing the calculation using two different datasets for emissions from soybean production, Ecoinvent v3.0 or Ecoinvent v2.2. Ecoinvent v3.0 includes improved estimates of emissions from land use change (Nemecek et al., 2014), and therefore produces lower estimates of emissions from recycling food waste as feed (which leads to avoided emissions from the production of conventional feed). Error bars show one standard deviation.

Land use change emissions are the largest source of greenhouse gas emissions associated with certain forms of animal feed, notably soybean meal (van Middelaar et al., 2013), yet are ignored in much of the literature on food waste disposal technologies (but see Tufvesson et al., 2013, van Zanten et al., 2014). In this study, we therefore used the most recent data available on land use change emissions for soybean meal (Nemecek et al., 2014), a major constituent of EU pig feed. This has a large effect on the modelled emissions from wet and dry feed (Fig. 5). This shows the importance of using updated data inventories for agricultural products, whose emissions vary over time and whose measurement is rapidly improving.

### 3.3 Wet vs dry feed

**Fig. 6:**
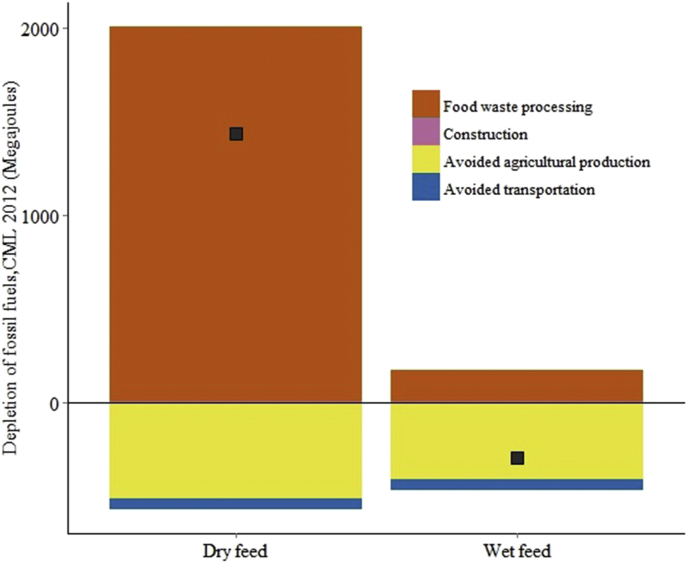
Fossil fuel use (MJ) in the production of wet feed and dry feed. The processing stage for dry feed has much higher fossil fuel use than wet feed because of the additional dehydration of food waste during production. Both avoid fossil fuel use associated with the production of conventional pig feed. The black points mark the net fossil fuel use per technology.

We evaluated two different technologies for recycling food waste as animal feed. We find that the processing of food waste into a sterilized wet feed has lower environmental and health impacts for all indicators, compared with processing into a dry pig feed. The difference between wet and dry feed results in large part from the higher fossil fuel inputs required to dehydrate municipal food wastes (Fig. 6). Municipal food wastes have a high water content (typically 65–80%; zu Ermgassen et al., 2016), and their dehydration to make dry feed requires gas and electricity (Table 5). This result does not, however, suggest that food waste feed should always be fed as a wet feed, because these two technologies may be suitable for different pig production systems. In South Korea, for example, dry feed is often produced in centralised facilities before resale and transport to farmers, while wet feed has a much higher water content and is therefore more expensive to transport. It is typically produced on or near to pig farms in order to minimise post-processing transport costs. The suitability of dry or wet pig feed depends in part on the proximity of pig farms to sources of food waste. For this reason, wet food waste feed, or “swill”, has long been a favoured pig feed for smallholder farmers (Westendorf, 2000). Most industrial pig farms in the UK currently use dried feeds; wet feeding is more common in other EU nations, such as the Netherlands, where it is favoured because it permits the use of wet agricultural wastes, such as distillery wastes or beet tails (van Zanten et al., 2014), and because of reported nutritional benefits of wet feeding (Brooks et al., 2001, Missotten et al., 2015).

### 3.4 Other species

Food waste can be fed to livestock other than pigs, including poultry, fish, and ruminants (Angulo et al., 2012, Boushy and van der Poel, 2000, Cheng et al., 2014). This study focussed on the use of municipal waste as pig feed because they have a long history of recycling waste into animal products (Fairlie, 2010), and because there are human health concerns with feeding food wastes which contain animal products to other livestock species, notably ruminants. The use of meat wastes in ruminant (cattle, goat and sheep) diets is banned in the EU because of concerns about Bovine Spongiform Encephalopathy (BSE), a disease that does not affect pigs, poultry, or fish (Andreoletti et al., 2007). Alternatively, food wastes can be fed to insects which may in turn be used as animal feed (van Zanten et al., 2015). This practice would be inherently less efficient than feeding food waste to pigs directly, and is also currently illegal, though there is an active campaign for the legalisation of the use of insects in animal feed (Searby, 2014).

### 3.5 Barriers to adoption

This study suggests that the use of municipal food waste as animal feed could reduce the impact of food waste disposal in the UK. This practice is currently illegal and there are a number of barriers to its adoption, both political and infrastructural.

Animal feeds are of course not only selected on their environmental merit. The re-legalisation of swill would require the confidence and support of the public, pig industry, and policy makers. Though heat treatment renders food waste safe for pig feed, there is some concern that the re-legalisation of heat-treated food waste feed might increase the risk of uncooked food waste entering the feed supply, potentially leading to disease outbreaks in livestock. If re-legalised, however, the potential benefits of using food waste as feed include reduced impacts on the environment, improved profitability for many farmers, and high meat quality and taste (for more detailed discussion see zu Ermgassen et al., 2016).

Food waste can only be used in animal feed if it is collected separately from other wastes and is sufficiently fresh. While this is the case in countries like South Korea and China (Chen et al., 2015), food waste collection in the UK is currently more variable. In 2013, separate food waste collection occurred in 95% of Wales, but only 34% of Scotland, 26% of England, and 4% of Northern Ireland (House of Lords, 2014). The potential use of food waste as animal feed is therefore not only a function of the availability of food waste, but its accessibility and quality. Where food waste is of poor quality or not adequately separated, it can be diverted to composting or anaerobic digestion, in line with the food waste hierarchy. It is promising that separate food waste collection in the UK increased from 15,000 tonnes in 2006 to nearly 350,000 tonnes in 2012 (DEFRA, 2015b).

## 4 Conclusion

While feeding municipal food waste to livestock is currently illegal in the EU, it is a common practice in many parts of the world, and there is growing interest in its potential use as a replacement for high-impact, high-cost conventional pig feed. This is the first study to compare the environmental impacts of recycling municipal food waste as animal feed with alternative disposal options in the EU. We used a holistic, hybrid LCA approach to compare four food waste disposal technologies in terms of 14 different environmental and health impacts and found that converting municipal food wastes into pig feed would lead to lower environmental and health impacts than processing waste by composting or anaerobic digestion – the UK government's currently preferred disposal options (DEFRA, 2015a). The widespread use of food waste as animal feed in the EU will require consumer and industry support, policy change, and investment in food waste collection infrastructure. Our results suggest that if these barriers can be overcome, the re-legalisation of food waste in pig feed could lead to substantial environmental and health benefits.

## Figures and Tables

**Table 1:** Environmental impact categories and the normalisation references used in this study (Benini et al., 2014). ​CTUh = comparative toxic unit for humans, CTUe = comparative toxic unit for ecosystems, and AE = Accumulated exceedance.

Impact category	Abbreviation	Method	Unit (characterised/normalised)	Normalization factor per person (domestic)
Climate Change	GWP	IPCC 2007	kg CO_2_-eq./mPE year^−1^	9.22E+03
Stratospheric Ozone Depletion	ODP	WMO 1999	kg CFC-11-eq./mPE year^−1^	2.16E-02
Human Toxicity, Cancer Effect	HT-C	USEtox	CTU_h_/mPE year^−1^	3.69E-05
Human Toxicity, non-Cancer Effect	HT-NC	USEtox	CTU_h_/mPE year^−1^	5.33E-04
Ionizing Radiation, Human Health	IR	Dreicer	kBq U^235^ eq./mPE year^−1^	1.13E+03
Photochemical Ozone Formation		ReCiPe midpoint	kg-NMVOCeq/mPE year^−1^	3.17E+01
Freshwater Eutrophication	FEP	ReCiPe midpoint	kg P-eq./mPE year^−1^	1.48E+00
Marine Eutrophication	MEP	ReCiPe midpoint	kg N eq./mPE year^−1^	1.69E+01
Freshwater Ecotoxicity	ET	USEtox	CTU_e_/mPE year^−1^	8.74E+03
Depletion of Abiotic Resources-Fossil	ADP-F	CML	MJ/mPE year^−1^	6.24E+04
Depletion of Abiotic Resources-Elements (Ultimate Base)	ADP-E	CML	kg Sb-eq./mPE year^−1^	1.01E-01
Acidification	AP	Accumulated Exceedance	AE/mPE year^−1^	4.73E+01
Terrestrial Eutrophication	TEP	Accumulated Exceedance	AE/mPE year^−1^	1.76E+02
Particulate Matter	PM	Humbert	kg PM_2.5_/mPE year^−1^	3.80E+00

**Table 2:** Municipal food waste composition data for the UK and South Korea.

		United Kingdom			South Korea
		(Zhang et al., 2013)	(Banks et al., 2011)	(WRAP, 2010)	(Kim and Kim, 2010)
PH		5.4	5	n.a	4.2
TS	%ww^a^	27.3	24.4	27.7	20
VS	%ww	25.4	22.3	23.35	14.7
Ash	%ww	1.8	2.1	2.0	5.3
CV	MJ/kg TS	21.1	21.2	26.53n	1.18–20.27
**Elemental analysis**					
N	%TS	2.9	3.2		3.6
C	%TS	49.7	50.3	49.32	51.0
H	%TS	6.4	6.3	6.5	6.0
S	%TS	n.a	0.2	0.4	0.2
O	%TS	34.7	31.7	37.1	39.2

a ww = wet weigh.

**Table 3:** AD digestate utilization efficiencies (Wallace, 2011).

	Unit	Value	Efficiency (%)
Readily available N^a^	kg/m^3^	5.94	34.5
Total phosphate (P_2_O_5_)	kg/m^3^	0.48	46
Total potash (K_2_O)	kg/m^3^	1.81	60

a 40% of the readily available content of nitrogen is lost during spreading.

**Table 4:** The 2010 UK electricity national grid (DECC, 2014).

Electricity sector	Amount (kwh)^a^	Percentage (%)
Hard coal	0.29	28
Hydropower	0.01	1
Natural gas	0.46	46
Nuclear	0.17	17
Industrial oil	0.02	2
Wind power plant	0.03	3
Biomass	0.04	4

a Total may not equal 1 kwh due to rounding.

**Table 5:** Life cycle inventory data of food waste management options.

		Materials	Unit	Animal dry feed^1^	Animal wet feed^1^	Anaerobic digestion^2^	Composting^2^
Input	Food waste		kg	1000	1000	1000	1000
	Corn starch		kg		250		
	Sawdust						
	Process water		kg	2.53		236	110.8
	Woodchip		kg				0.31
Energy	Gas			32.5			
	Electricity (see table)		kwh	24.6	3.86	65	5.78
	Diesel		kg		2.47	0.081	3.29
Product			kg	130	430	Digestate (659) Electricity (260 kwh)	659
Waste	Wastewater		kg	640	564	320	
	Screening/rejected materials		kg	60	30		30
Process air emissions							
	CO_2_		kg	8.7E+01	7.9E+00	2.6E-01	1.1E+01
	CH_4_		kg	1.6E-03	3.2E-04	3.4E-02	4.8E-03
	N_2_O		kg	1.6E-04	6.4E-05	1.9E-02	2.7E-02
	NO_x_		kg	2.3E-01	2.1E-02	4.4E-02	1.0E-01
	CO		kg	3.1E-02	1.6E-03	1.5E-03	5.9E-02
	MVOC		kg	7.8E-03	0.0E+00	2.4E-02	6.0E-03

Sources ^1^: (Kim and Kim, 2010) and ^2^ (Hall et al., 2014).

**Table 6:** Characterised results for four recycling technologies. Lower values in each row are standard deviations. All differences between technologies are statistically significant (p < 0.01), except those marked by a^#^. Abbreviations are listed under Fig. 3.

Disposal technology (Units)	GWP (kg CO_2_e)	ODP (kg CFC-11e)	HT-C (CTU)	HT-NC (CTU)	IR (kg U235e)	POF (kg NMVOC)	FEP (kg Pe)	MEP (kg Ne)	ET (CTU)	ADP-F (MJ)	ADP-E (kg antimony-e)	AP (AE)	TEP (AE)	PM (kgPM2.5e)
Dry	3.96E+01	3.08E-06	−1.30E-07	−9.37E-05	3.65E-01	−9.73E-02	−2.17E-02	−1.38E+00	−2.20E+02	4.03E+03	7.96E-05	−6.48E-01	−3.25E+00	−2.45E-02
	3.92E+01	1.37E-06	7.05E-08	1.66E-05	8.38E-01	6.57E-02	3.54E-03	2.13E-01	3.41E+01	5.92E+02	1.33E-04	1.51E-01	6.39E-01	9.71E-03
Compost	2.80E+02	1.87E-05	1.06E-06	1.17E-04^#^	3.45E+00	6.69E-01	2.86E-02	1.93E+00^#^	2.95E+02	3.43E+03	1.25E-03	1.59E+00	6.97E+00	7.62E-02
	2.67E+01	3.13E-06	1.59E-07	2.63E-05	7.66E-01	1.09E-01	6.36E-03	3.91E-01	6.11E+01	6.12E+02	2.14E-04	2.74E-01	1.15E+00	1.51E-02
AD	3.80E+01	7.34E-06	9.34E-07	1.18E-04^#^	7.90E+00	5.70E-01	2.69E-02	1.91E+00^#^	3.03E+02	−1.73E+03	1.32E-03	2.05E+00	9.45E+00	8.90E-02
	7.34E+01	2.03E-06	1.83E-07	2.64E-05	1.67E+00	1.72E-01	6.84E-03	3.93E-01	6.11E+01	1.25E+03	2.42E-04	3.83E-01	1.25E+00	2.15E-02
Wet	2.09E+00	−2.95E-06	−2.81E-07	−1.02E-04	−2.37E+00	−2.67E-01	−3.00E-02	−1.77E+00	−2.76E+02	1.80E+03	−5.17E-04	−1.00E+00	−4.38E+00	−5.45E-02
	3.56E+01	2.38E-06	1.69E-07	3.47E-05	1.03E+00	1.42E-01	8.61E-03	5.26E-01	8.24E+01	6.00E+02	2.80E-04	3.70E-01	1.54E+00	2.03E-02
